# Discovering what patrons value in a consumer health library service using laddering interviews

**DOI:** 10.5195/jmla.2023.1495

**Published:** 2023-04-21

**Authors:** James Michael Lindsay, Courtney Wombles, David Petersen

**Affiliations:** 1 jmlindsay@utmck.edu, Head of Collections & Access Services, Associate Professor, Preston Medical Library University of Tennessee, University of Tennessee Medical Center, Knoxville, TN.; 2 courtney.wombles@lmunet.edu, Medical Librarian, Lincoln Memorial University, Harrogate, TN.; 3 dpetersen@utmck.edu, Assistant Professor, Research & Learning Services Librarian, Preston Medical Library, University of Tennessee Graduate School of Medicine, Knoxville, TN.

**Keywords:** Laddering interviews, customer value determination

## Abstract

**Background:**

Librarians at Preston Medical Library sought to understand whether marketing research techniques could be adapted to libraries to better understand what patrons value. Specifically, this study sought to learn why patrons continue using a consumer health information service, develop insights to improve the service, and a methodology to use with other patron groups.

**Case Presentation:**

Librarian researchers conducted customer value research using laddering interviews, an interview technique utilized in marketing research to learn users' goals in using a product or service. The PML research team interviewed six frequent users of a medical library's consumer health information service. Researchers conducted laddering interviews, covering patron views of basic attributes of the service, leading on to consequences of their interaction with it, and finally discussing what they hoped to achieve in using the service. The results were visualized in customer value hierarchy diagrams, graphically showing relationships between valued attributes of a product or service, how the patron used it, and how that helped patrons achieve goals. This allowed the research team to identify which features of service contribute the most to patron satisfaction.

**Conclusion:**

Customer value learning utilizing laddering interviews enables librarians to see their service through the patrons' eyes, focusing on those aspects of the service that they view as most important. This study allowed librarians to learn that users desired to feel more in control of their health and gain peace of mind by obtaining trusted information. The library's work in providing information leads to self-empowerment for these patrons.

## BACKGROUND

The Consumer and Patient Health Information Service (CAPHIS) has been offered to patrons of Preston Medical Library (PML) and the Health Information Center since 1993. Librarians research answers to patron inquiries at no cost regarding medications, conditions, and diagnoses. Users include hospital patients, patient family members, public patrons, and hospital staff members. The service is accessible in-person, directly from patient room televisions, or by email, telephone, or web form. Upon receiving each request, library staff review the inquiry and provide the needed information. Requests made in-person are frequently hand delivered; however, for patrons calling or emailing, results are sent via email or postal mail depending on patron preference.

The library serves a varied set of patrons, including the University of Tennessee Graduate School of Medicine (658 FTE), part of the University of Tennessee Health Science Center. Further, the library is located within the University of Tennessee Medical Center, a single-hospital health system (698 staffed beds) independent of the University. Staff and patients in both institutions use the service, but CAPHIS's reach extends into the local community, due to outreach efforts conducted over several years [1]. The library benefits from word-of-mouth advertising [2], and librarians have presented to community organizations and public libraries in the area. PML librarians sought to learn what motivated these users to pursue the service, and what they wanted to receive. While satisfaction surveys have been used consistently for much of the service's history, librarians did not know what motivated repeat patrons to seek out information from the library [1].

**Figure 1:**
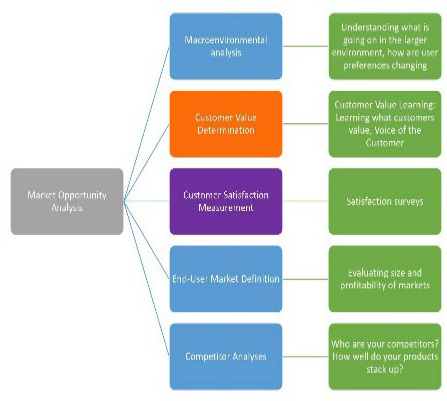
Context of Customer Value Determination

In addition to learning what motivated repeat users of the CAPHIS service, PML researchers also sought to learn if market research techniques could be applied in a library context. Specifically, the research team implemented customer value learning, a subset of market research focused on discovering problems customers desire to solve using a product or service and how they see it helping them achieve a desired state [3]. Integrating information from multiple sources to produce a clearer picture of customer needs and wants, customer value learning, or customer value determination, is one part of a larger, integrated set of activities known as market opportunity analysis (MOA) (Figure 1). The research team hypothesized that the CAPHIS service and the library more broadly would benefit from this more systematic approach by understanding patron desires and expectations, informing customer satisfaction measurement. This understanding facilitates the creation of satisfaction surveys that measure service performance against patron expectations, establishing a patron-centered standard against which to measure the service. This would go beyond assessing operational efficiency and effectiveness, guiding library promotional efforts for services by aligning the library's messages with user's actual values, rather than relying on library staff's intuitions or users' stated information needs. In other areas of library work, specifically reference, we learn as librarians to dig a little deeper to discover what users really want, which can be quite different from their initial query. A simple request for “articles on Diabetes” from a physician could yield, with probing, to be a request for research to update a pathway on treatment for Diabetic Ketoacidosis. This same incisiveness can be applied to our approach to promoting library services.

Figure adapted from: Woodruff RB, Gardial SF. Know Your Customer: New Approaches to Understanding Customer Value and Satisfaction. Malden, MA: Wiley-Blackwell, 1996.

Several approaches have been used by market researchers in private industry to learn what customers value. These include focus groups, observational studies, and in-depth interviews [4]. The PML research team chose the Laddering interview technique, a type of in-depth interview. Laddering can be conducted at small scales, minimizing resources required while allowing for unexpected insights. Laddering interviews have been utilized in business research studies since the 1980s, with Reynolds and Gutman publishing a seminal study that detailed the concept of laddering and describing how it could illustrate the consumer process [5]. Laddering interviews have been utilized to understand consumer insights [6], assess student opinions of service quality in higher education [7], and probe the desired qualities and behaviors of physicians [8]. Engagement with laddering interviews within library science literature has been relatively small, with only five total studies published in recent years and very few published by North American authors [9–13].

Laddering interviews are conducted one-to-one; in customer value learning research settings, the researcher interviews a single patron. The researcher elicits patron views on attributes of a service, such as the format in which information is sent, or how quickly it was received. The researcher then proceeds to how patrons interacted with a service. In asking about the consequences of the patron's interactions with the service, the researcher witnesses their reactions to specific aspects of the service, learning what worked well for them and what did not. By probing, the interviewer learns what the patron feels and wants to happen when they use the service. Following analysis, the data is assembled in a customer value hierarchy. This graphically represents patron views on attributes, experiences interacting with the service, and goals for using the service.

## CASE PRESENTATION

**Figure 2:**
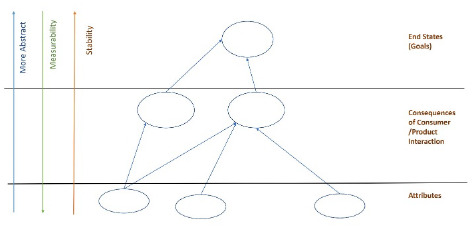
Customer Value Hierarchy – Model

**Figure 3:**
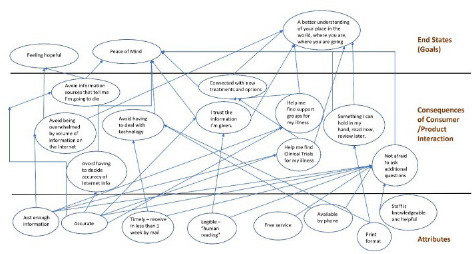
Customer Value Hierarchy for “Joy”

The PML research team conducted six laddering interviews, evaluating goals and experiences of CAPHIS users. For each of the six total interviews conducted [14–19], customer value hierarchies were built according to the template (Figure 2). In constructing customer value hierarchies, the PML research team began with interviewees' impressions on the basics of the service, described as attributes. These were briefly described, using their own words when possible, and added at the attribute level on the diagram (Figure 3). Moving up the customer value hierarchy, participants were asked about their interactions with the service. When asked about this, our team was able to link these initial impressions regarding attributes to the consequences of their interactions with the service. Linking attributes to consequences occasionally required interpretation from the interviewer. Often, multiple attributes will work together to provide the desired value. Finally, the interviewer addressed the goals and values of the user or the metaphorical top rung of the laddering interview. We wanted to know what they expected to learn, and how they wanted to feel about that.

The lead author conducted the initial 2 interviews as a convenience sample to fulfill the requirements of an MBA marketing assignment. Upon completion, the project was expanded, involving other library staff, resulting in an additional 4 interviews. The CAPHIS service maintains a database of all users, which was queried to obtain a list of interview candidates. The PML research team targeted repeat users; 17%, or 279 users out of a total of 1,676 since the service's inception, have used the service more than once. The PML research team downloaded the list, sorting based on frequency of use, and contacted the 10 candidates who had used the service most frequently.

The initial two interviewees were long time CAPHIS users who were personally recruited by the lead author via telephone. The University of Tennessee Graduate School of Medicine's Institutional Review Board provided exempt approval for this initial study and for the later expanded study. The first author received training in conducting these interviews in a marketing lecture [4], participated in an in-class simulation, and received an interview guide. Both interviews were conducted via telephone, and call recording apps were used with subject permission to document interviews. Questions specific to the service were added to those provided in class. Subjects were asked about preferred format for receiving information, timeliness of service response, the quality of information received, and interactions with library staff. Open-ended questions encouraged patrons to share ideas the research team might not have anticipated when designing the interview protocol. The results of this initial study provided a proof of concept that could be used in expanding the study to other users, developing a larger scale qualitative methods research study.

Following the initial interviews, the first author worked with colleagues on a plan to expand the study. The research team met with a qualitative research expert to develop an expanded research plan, which was approved for use by our IRB. These plans resulted in the development of an interview guide (Appendix 1) and a plan for contacting users of the service. We prepared a letter, which was signed and then sent by postal mail under the library director's letterhead to selected users. Users responded by telephone to the interview invitation, and an appointment was made upon acceptance. From the initial mailing, there were three respondents. PML researchers then repeated the process to contact another ten candidates, yielding another additional interview, for a total of four new interviews. Combined, a total of six interviews were conducted, counting the initial study. The interviewers utilized the video conferencing software Zoom to schedule appointments, conduct interviews, and record audio of each interview. Interviews were recorded with the patron's consent, allowing the interviewer to listen later and assemble customer value hierarchies, graphically representing the results.

Library staff received training in conducting the interviews. They administered the laddering interviews using an interview guide, including a pre-determined set of guiding questions. After explaining the study, ensuring confidentiality, and encouraging interviewees to speak openly and honestly, the interviewer began by asking the patron about basic attributes of the consumer health information service. After probing users' interactions with the service, they were asked to assess how using the service made them feel before and after contacting the library. Following the interviews, our team reviewed the recordings and notes and constructed customer value hierarchies. Librarians designed a template, seen in Figure 2 that was used to diagram the interviews based on the marketing lecture and text [3, 5].

These interviews allowed our team to learn what our participants valued; they noted the thoroughness, accuracy, and accessibility of the information provided. As noted by “Joy,” she received “just enough information”, that was “legible” (understandable to a non-healthcare professional) (Figure 3) [14]. Moving up the customer value hierarchy and describing their interactions with the service, “Joy” was able to avoid dealing with technology and difficulties in judging information quality online. She felt encouraged to ask additional questions; others, such as “Keith,” obtained faster answers to questions [18]. Interactions with library staff were also mentioned frequently as a strength of the service. “Joy” reported that staff were “knowledgeable and helpful;” “Dee” noted that the library “isolates irrelevant information,” so she obtains needed data [14, 15].

Based on these hierarchies, the research team identified three major themes across the respondents: satisfaction with receiving printed information, an appreciation of how the service saved users' time, and a feeling of relief and comfort from the knowledge they received.

### Receiving printed information

As noted by “Joy,” having printed material was something she could “hold in my hand, read now, review later (Figure 3).” “Dee” referred to it as “facts in my hand that serve as leverage [15].” “Betty” viewed it as “convenient” to receive information in this manner as she could no longer drive [17]. “Keith” appreciated how organized the information was, and how he would receive a brief overview or facts over the phone, with the “expanded version” in the mail (see supplemental files for additional hierarchies) [18].

### Time-savings

The time users saved by contacting the library was also valued by interviewees. “Keith” described what he received as “concentrated information” that “saves me time,” which helped him “deal with health issues in a more informed and quicker way, which is important when you are ill.” In addition, “Robert” mentioned that requesting research from the library was “easier than searching on my own [19].”

### Feelings of relief and comfort

Many users described a feeling of relief; “Joy” reported “feeling hopeful” (Figure 3), “Dee” described “being able to stand on my own two feet.” Others described being able to better adjust to their health challenges [18] and hoped that the service would continue [17]. One interviewee even wanted to include the library in his will. For these interviewees, seeking out health information helped them feel more empowered, comforted, and in control.

While there were similarities in the goals of interviewees, there were also many differences in their needs. Some were comfortable using computers but not comfortable doing their own research; others were comfortable using medical texts but lacked transportation and access to trusted materials. From CAPHIS satisfaction surveys, our research team knew that 80% of users were aged 60 or more; some frequent users were younger, however. Most users were asking questions for themselves, but some requested research on behalf of friends and family.

The interviews conducted with “Dee” and “Joy” illustrate that learning more about their conditions helped both women to be more active participants in their own healthcare [14–15]. Both wanted to gain a better understanding of their conditions, peace of mind, and a feeling of hope by knowing more. These individuals also expressed satisfaction with the CAPHIS service by saving their time, reducing the volume of information to filter, and the quality of provided information. The End States (goals) differed between the hierarchies with “Joy” having reported feeling “hopeful” and having “peace of mind” while “Dee” emphasized feeling “better informed.” As noted by “Joy” in the interview diagrammed in Figure 3, she reported “a better understanding of your place in the world, where you are, where you are going” [14]. The goal for the marketer of any product or service is to provide value [3,4]. This is done by learning to “identify”, “choose”, “provide”, “communicate” and “assess the delivered value.” [3,4]. While both individuals approved of and found value in the CAPHIS service, they did so for different reasons.

## DISCUSSION

This study allowed the library an opportunity to assess the value of the laddering technique, a method frequently used in business research but rarely used in libraries. This research can be a starting point for rethinking how libraries evaluate and improve services. The laddering technique allowed interviewers to discover CAPHIS user goals. Utilizing the service allowed users to avoid information overload, technology anxiety, judging online information quality, and sensationalistic sources that generated anxiety. Additionally, users noted library staff encouraged users in their inquiries, making them feel at ease to ask additional questions.

As the study progressed, many lessons were learned that would be useful for libraries attempting this type of research. Although more coaching was required of the respondents, Zoom allowed for interviews to be easily scheduled and recorded. Respondents did not need a computer to access this feature, and recordings could be sent to us for later analysis. From a technical perspective, using Zoom to record the interviews was far easier than using phone recording apps. Phone recording apps required adding a second call onto the first one, which simply failed in the second interview, requiring the first author to depend on interview notes. With Zoom, however, the interviewer was able to schedule the interview and make a quick call to the interviewees to share the Zoom call number. No technical issues were experienced in any of the ensuing interviews.

One issue noted was that not all library staff were prepared when patrons called the library to set up an interview; ensuring that respondents feel welcomed when they respond to the interview request is critical to success. The interview guide was a crucial tool in the study but could have been improved before submission to the IRB. Although it was prepared with the aid of a qualitative research expert, the laddering concept was not adequately specified in the guide. While student research assistants were trained in using the technique, in retrospect the training and guide could have been more effectively harmonized to reinforce the technique. At times, the greatest challenge in conducting these interviews was to stay out of the patron's way. There can be a fine line between asking probing questions and leading interviewees. As noted in a University of Tennessee marketing professor's lecture regarding depth interviews, the aim of the interview “is not to add your own opinion or comments” [4].

Another important lesson learned was that the IRB filing for the study needed broader contact parameters. Out of 20 letters sent, there were only four additional respondents, a response rate of only 20%. The PML research team over-estimated the number of respondents there would be, assuming regular users of the service would be more likely to respond to a request by mail rather than telephone. A goal response rate for mailed surveys of 30% had historically been used as a rule of thumb at the researchers' institution, but the team was unable to locate a peer-reviewed source validating this specific figure. Additional research showed that acceptable figures for response rates can vary from 25-75%, and researchers at this institution had recently been advised to pursue response rates of 60-80% to reduce non-response bias [20,21]. However, the guidelines our team was most familiar with pertained to quantitative surveys rather than qualitative research. In qualitative research, the goal is to reach saturation [4,22]. Essentially, this means that the research continues until you cease learning anything new. Our team realized that by this metric at least, we still had more to learn. Future studies will include broader contact parameters and a revision of the interview guide.

Preston Medical Library, like many other institutions, utilizes user surveys to assess user satisfaction with its consumer and patient health information service. This study allowed us to look beyond satisfaction surveys to learn about the motivations and preferences of users. Users of the service derive comfort and self-confidence from learning more about their health and being better able to deal effectively with health issues. These insights will help the library in crafting messages for users. More than simply describing services to patrons [23], action-oriented messages will be created to invite users to gain knowledge and take charge of their health. The library's satisfaction surveys have improved over time; however, understanding patron goals provides a clearer target for librarians. By measuring progress in helping users to gain control of their health and their lives, librarians can demonstrate the value of their services to those that matter most. Using laddering interviews to perform customer value research is an adaptable, low-resource technique that can be used by libraries of varying sizes to gain crucial insights about what users value in library services.

## Data Availability

Data associated with this article cannot be made publicly available because they contain personally identifiable information. Access to the data can be requested from the corresponding author and may be subject to IRB restrictions.

## SUPPLEMENTAL FILES

**Appendix A:** Interview Guide-Blinded
