# Supplementary material for: Discovering what patrons value in a consumer health library service using laddering interviews
Source: J Med Libr Assoc. 2023 Apr 21;111(1-2):612–7. doi: 10.5195/jmla.2023.1495 (PMC10259625; doi:10.5195/jmla.2023.1495)
Supplement: Supplementary file 1 — Appendix A: Interview Guide-Blinded [file jmla-111-1-2-612-s01.pdf]

Appendix A

Patient Value and the Library Consumer Health Information Service: A Qualitative Study

Interview Guide (DRAFT):

Introductory Script:

Thank you, _________________, for agreeing to talk with me today. My name is ________ and I am a librarian, doing this research for xx library.

I have called you today because we are working on a research project that involves talking with individuals who have used the library's Health Information Service. Through interviews, we are trying to understand why patients use our service more often than others. We hope to be able to understand how to increase the number of these users.

I want to encourage you to speak openly and honestly about your opinions, ideas, and experiences. There are no right or wrong answers to the questions I will ask.

The interview should take about one hour. I will be audio recording this interview because I want to make sure that I capture your responses accurately.

I want to be sure that you understand that all of your responses will be kept confidential. This means that everything you say will only be shared with the research staff. We will ensure that any information we include in our reports does not identify you as a participant. If you are uncomfortable at any time, please let me know and we can stop the interview, or take a break. You are also welcome to skip any question that you are uncomfortable answering.

Your participation in this study is voluntary. You may decide not to participate without penalty. If you withdraw from the study before data collection is completed, or if you decide you don't want your interview to be included, your data will be destroyed.

If you have questions at any time about the study or the procedures, you may contact me, xxx, at xxx, or by email at xxx. You can also contact my research supervisor, xxx at xxx Library (xxx)xxx-xxxx, or by email at xxx. If you have questions about your rights as a participant, contact the xxx Institutional Review Board at (xxx)xxx-xxxx.

What questions do you have about what we've talked about so far?

Okay, let's begin.

Interview Questions:

1. Tell me about your experiences with our service.

Potential Prompts under this question:

How did you find the usability of our resources?

Does the format in which you receive information matter to you? (i.e., print, electronic, email)

Tell me some things about our service that you find useful.

Tell me some things about our service that you feel may need improvement.

2. What or who led you to using our service?

Potential Prompts under this question:

How do you access our service (i.e., phone, home computer, work computer, public computer)

Do you own a computer?

When did you begin using our service?

Are there areas of our service that you feel should be more user-friendly?

3. Think back to a recent experience with our service. What information were you looking for? On a scale of 1-10, with 10 being the easiest, how easy was it to find the information you were searching for? Why was it that?

Potential Prompts under this question:

How did you feel before you came to us?

How did you feel after?

What are your thoughts on how long it took to receive information?

How did you feel about the quality of the information you received? Did you view it as credible or trustworthy?

Did you receive information you don't think you could have found on your own?

Was the amount of information you received just right, too much, or not enough?

If our service was not available to you, where would you have looked to find the information you needed on an alternate website or resource?

4. Tell me about a time when you could not easily find information on our service.

Potential Prompts under this question:

Where did you go to find the information you needed?

What can we do to improve our service so this type of instance does not happen again?

5. Tell me why you continue to use our services.

6. What else would you like to add about our services?
